# COVID-19: A Neoliberal Nirvana?

**DOI:** 10.1007/s10612-022-09652-x

**Published:** 2022-07-04

**Authors:** Richard Watermeyer, Rille Raaper, Margarida Borras Batalla

**Affiliations:** 1 Centre for Higher Education Transformations, University of Bristol, Bristol, UK; 2 School of Education, Durham University, Durham, UK; 3 School of Education, University of Bristol, Bristol, UK

## Abstract

The COVID-19 pandemic has profoundly impacted the operation of universities around the world. A transition to online platforms and remote forms of working as a consequence of national lockdown measures and campus closures has produced new labour challenges for academic faculty. This article makes use of 12 months of reporting from the academic trade press related to the experience of the pandemic in the UK higher education sector. Accounts published within *Times Higher Education* signpost the accelerating and accentuating effects of COVID-19 as it relates to universities’ neoliberalization; corporate managerialism within UK universities; and academic work precarization and work-based inequities.

## Introduction

The COVID-19 global pandemic has been experienced as a force of unparalleled disruption to the operational dynamics of universities around the world (*cf*. Agasisti and Soncin 2021; de Boer 2021; Metcalfe 2021; van Schalkwyk 2021). Concern for the future of universities is amplified by its core constituency of academics who are fearful of role invalidation and jobs obsolescence as a consequence of ubiquitous digital disruption and migration to online platforms accelerated by campus closures. The impact of digital transitioning, and the potential even of a permanent digital resettlement (Watermeyer et al. forthcoming)—linked to market reorganization and the opening of new market opportunities for higher education providers—is also attributed to a re-rationalization of universities’ core investments, labour streamlining, and cost-cutting exercises that threaten not only individual livelihoods but the sustainability of academic programmes and whole departments (Blackmore 2020; Raaper and Brown 2020). Such fears are exacerbated by findings, from a recent large-scale survey of UK academics, that detect a ‘woeful state of management and governance in the UK HE sector’ which has culminated in “an acute situation of endemic bullying and harassment, chronic overwork, high levels of mental health problems, general health and wellbeing problems, and catastrophically high levels of demoralization and dissatisfaction” (Erikson et al. 2020: 15).

Academics’ occupational precarity is hardly, however, *sui generis*. In fact, it is intimately tied to a full if recent history of financialization for the UK’s higher education sector, which features universities’ evolution into (quasi)market entities, and a structural shift in the governance of universities from a model of academic collegialism to corporate managerialism; rife among which is a fatalistic discourse of academics ceding rights of critical freedom and autonomy and their capitulation to performative ritualism and neoliberal governmentality (Olssen and Peters 2005; Raaper and Olssen 2016). Such trends have spawned an abundance of critical research that has sought to problematize the transformational effects of organizational and cognate ideological shifts on academic lives. Centre stage is a concern of how twinned cultures of managerialism and audit, undergirded by the introduction of new public management technologies and a commitment to performance enhancement through market competition, have been detrimental to the health and wellbeing of academics and more widely injurious to research and pedagogical praxis (Tamboukou 2012; Thornton 2013)—while ironically making no discernible contribution to academics’ productive capacity and output. We may point for instance to the carceral logic (*cf*. Wacquant 2010) that supports neoliberal technologies of governance, or as we prefer, *control*, like the UK’s Research Excellence Framework (REF)—a performance-based research funding system through which universities compete for positional goods of government finance and scholarly prestige—that incentivize excellence-making at the cost of exclusion (Watermeyer and Olssen 2016) or the dispossession of professional identity (Watermeyer and Tomlinson 2021). The ‘violence of neoliberalism’ (Collins and Rothe 2019) upon university communities in the UK is also observed as a trend of self-responsibilization and, relatedly, precarization, for academics in matters of personal, financial, and occupational welfare. Such a trend allied to the seeming collapse of a duty of care among university and higher education sector leaders has brought about a level of toxicity to the neoliberal university (Smyth 2017) which is responded to with large scale industrial action (*cf*. UCU 2020a) and, in the most desperate of cases, with academics taking their own lives (*cf*. Parr 2014; Pells 2018).

In the present milieu of what we have elsewhere observed as a condition of ‘pandemia’ (Watermeyer et al. forthcoming), such historical concerns of the corrosive and debilitative influence of ‘new managerialism’—and its response, ‘competitive accountability’ (Watermeyer 2019)—become further problematic where universities are crisis managed and led according to a blueprint for damage limitation in which concern for academics may be an absent or else unacknowledged priority. ‘Soft’ impacts to academic welfare caused by the pandemic, we hypothesize, are thus in crisis mode, over-shadowed or made invisible by ‘hard’ impacts to universities’ economic stability. The pandemic may thus provide cover to many of the associated ills of universities’ neoliberal reforms and equally provide legitimacy to their neglect, which accordingly inspired our motivation to elucidate and cohere a narrative record, spanning the last, or be that *first* 12 months of COVID-19, that represents the ongoing transformation of the university and academic life in extraordinary times, if not quite so unprecedented conditions.

In what follows, we consider the intersection of the COVID-19 global pandemic as a source of profound societal disruption, with UK higher education as a site of equally severe systemic turbulence, change and resistance to change. We seek a window on to the extent of the pandemic’s disruption to higher education in the UK and the degree to which it may be seen to arrest or otherwise accelerate recent trends of neoliberalization that are blamed for the overall deterioration of academics’ professional (and personal) lives. We use accounts published from the UK academic trade press—Times Higher Education—to fulfil this aim and consider the susceptibility or else immunity of recent and ongoing higher education reform to the pandemic’s effects, by analysing representations of academics’ experiences of institutional responses to the incursions of COVID-19. Much, therefore, of our discussion is focused on understanding not just the impact of the pandemic on academics’ institutional lives, but the pandemic as an accelerator of labour-based transformation that deepens existing inequities, and which is further injurious to their occupational welfare.

## Research Approach and Methods

The study we report centred on the role of the UK academic trade press in the coverage of academic work and experience during the COVID-19 pandemic. We focused on *Times Higher Education* (THE)—the prestigious UK-based academic trade press specifically focused on news and issues related to higher education—to understand the ways in which neoliberal higher education policy drivers intersect with the pandemic to produce particular types of academic representations. Our use of THE follows the same rationale offered by Gewirtz and Cribb (2013: 60) in that “the publication itself symbolizes and has been an active participant in some of the key transformations in recent UK HE... and can be seen as representative, and in some ways constitutive, of that changing character”. In the latter instance, we observe THE wearing the same Janus-face borne by universities as (quasi)market competitors, where it operates dichotomously as a challenge to higher education’s neoliberal reforms yet also as an embodiment of neoliberal values—illustrated most emphatically by its vanguard role in the business of university rankings (*cf*. Stack 2020). Analogously, we find THE’s discriminatory bite tamed by its indulgence of celebratory spectacle such as its annual awards ceremony: a wellspring for higher education marketeers.

THE is published on a weekly basis, including a hard copy and online media output. Most British universities have purchased an institutional license to the magazine, making it the most widely read academic trade media outlet among higher education professionals and academics in the UK. It has a strong global facing, including the coverage of global higher education news and running of THE World University Rankings.^1^ According to THE website, the magazine has over 380,000 weekly readers, and 24 million visitors use the website every year (see THE n.d).

The data collection involved the period of 23 March 2020, as the start date of the first national lock down in the UK, to 12 February 2021, the end date of the project. We used the search engine on THE website, employing the following keywords: ‘academic*, ‘employee*’, ‘researcher*’. We applied broad keywords to capture all articles related to academic work. After the screening process, we identified 64 THE articles that addressed the impact of the COVID-19 pandemic on academic work and university practices, from out of which 51 articles were chosen for analysis. The outputs that lacked the relevance to the UK higher education context or addressed student issues were excluded from the sample.

The articles were downloaded and analysed based on a thematic analysis method (Braun and Clark 2006, 2013). By identifying core themes, the aim was to examine ‘underlying ideas, assumptions, and conceptualizations—and ideologies—that are theorized as shaping or informing the semantic content of the data’ (Braun and Clarke 2006: 84). The coding process was organic (Braun and Clark 2006), following five broad phases of analysis: (a) familiarizing with the data; (b) generating initial codes; (c) searching for themes; (d) reviewing the themes; and (e) defining and naming the themes (Braun and Clark 2006). When developing themes, we applied both deductive and inductive thematic analyses (Braun and Clarke 2006, 2012, 2013) to enable codes to emerge from THE articles analysed (stage 1) as well as using existing research on the neoliberalization of higher education and academic work as a lens through which to identify further themes (stage 2). Guided by deductive reasoning, the analysis process started with a broad pre-set understanding of neoliberalism as presented earlier in this paper. Neoliberalism in this project is approached as ‘a theory of political economic practices’ aimed at transforming the economic order through free market, entrepreneurial freedoms and property rights (Harvey 2005: 2). Through its operation, however, neoliberalism also becomes a mode of governance that alters the work and individual wellbeing which in a higher education context reflects an increasing use of performance indicators and managerialist practices (Olssen and Peters 2005). The theory-driven approach to analysis allowed us to identify themes related to marketization, managerialism, workload, and casualization. These themes were clearly evident in the dataset; however, it is important to note that through inductive analysis they were refined (see Table 1). The inductive approach therefore enabled us to detect an interaction between different themes and theoretical ideas (Braun and Clarke 2006, 2012) where issues related to workload and wellbeing, for example, became interconnected, resulting in a coherent single theme. Furthermore, the inductive approach provided an important space for unexpected or at least less expected themes to emerge such as the opportunities of COVID-19 in higher education as well as the issues related to gender and ethnic inequalities.

**Table 1:** The article themes

Theme	Frequency of mention (articles)	% (total 51)
*Workload and wellbeing*	34	67
*Workforce casualization and precarity*	22	43
*Intensified managerialism*	14	27
*Gender and ethnic inequalities*	12	24
*Opportunities of COVID-19 in higher education*	9	18
*Marketization of universities*	9	18
*Other*	2	4

The themes covered within the final sample of 51 articles are presented in Tables 1 and 2. The authors included THE reporters and editors (31), as well as academics (9), university senior management (3) and a small number of other specialists and PGR students (see Table 2). While many of the articles had a global focus, their relevance to the UK higher education context was evaluated, to assure that the authors were either from the UK, or that the issues discussed applied to a variety of national settings, including the UK (see Table 2).

**Table 2:** The article sample

	Publication date	Title	Section	Focus	Primary author background
1	23/03/2020	Coronavirus: King’s president to take 30 per cent pay cut	News	UK	Reporter
2	03/04/2020	Reacting to COVID-19 by slashing fixed-term staff would be a disaster	Opinion	UK	Professor
3	04/04/2020	We’re managing to be academics and primary school teachers	Blog	UK	Senior Lecturer
4	16/04/2020	Adequate is enough—and some days we won’t manage even that	Opinion	Global	Social Psychologist
5	20/04/2020	COVID-19: universities treating staff in ‘vastly different ways’	News	UK/USA/AUS	Reporter
6	20/04/2020	UK university staff ‘mental health crisis not getting better’	News	UK	Reporter
7	23/04/2020	UK universities ‘face £2.6bn coronavirus hit with 30 K jobs at risk’	News	UK	Reporter
8	07/05/2020	Roehampton is ‘first UK university to announce crisis job severances’	News	UK	Reporter
9	15/05/2020	Women in science are battling both COVID-19 and the patriarchy	Blog	Global	Unknown
10	18/05/2020	Most early career academics face funding cliff edge, survey suggests	News	UK	Data Editor
11	26/05/2020	Cambridge v-c: crisis could lead to job cuts in ‘worst-case scenario’	News	UK	Reporter
12	28/05/2020	Pandemic throws spotlight on HE employment practices	News	UK/USA/AUS	Data Editor
13	09/06/2020	Imperial COVID-19 researchers criticize plans to cut 75 ICT jobs	News	UK	Reporter
14	11/06/2020	HE financial crisis risks ‘lost generation of researchers’	News	Global	Data Editor
15	12/06/2020	Three challenges facing academic research during the COVID-19 crisis	Blog	UK	Director of Research
16	19/06/2020	Goldsmiths staff ‘at breaking point’ on job losses and racism claims	News	UK	Reporter
17	25/06/2020	The disproportionate effect of COVID-19 on women must be addressed	Opinion	UK	Master of Churchill College
18	25/06/2020	Pandemic lockdown holding back female academics, data show	News	Global	Reporter
19	25/06/2020	Juggling childcare with academia: female experiences in lockdown	News	Global	Reporter
20	25/06/2020	THE Leaders Survey: Will COVID-19 leave universities in intensive care?	Features	Global	Features and Opinion Editor
21	30/06/2020	COVID-19 is no longer a short-term crisis for higher education	Blog	Global	Director of Lab
22	01/07/2020	Solidarity with contingent faculty entails more than signing statements	Blog	Global	Head of School
23	06/07/2020	COVID-19 crisis could bankrupt a dozen UK universities, IFS warns	News	UK	Data Editor
24	09/07/2020	Bonfire of casual contracts ‘a huge setback’ for racial equality	News	UK/USA/AUS	Reporter
25	16/07/2020	Childcare the key limit on scientists’ pandemic working hours	News	USA/EU	North America Editor
26	27/07/2020	UK union fears thousands of job losses as casual contracts axed	News	UK	
27	31/07/2020	COVID-related exceptions from REF submission rules announced	News	UK	Reporter
28	09/08/2020	The ‘baby penalty’ was not born with the coronavirus	Blog	Global	News Editor
29	12/08/2020	Will COVID kill off the teaching-research employment model?	News	UK/AUS	
30	29/08/2020	More than 350 jobs at risk at London university	News	UK	Lecturer
31	07/09/2020	Academics whose institution has no post-COVID future should be laid off	Blog	Global	Asia Pacific Editor
32	12/09/2020	COVID-19 is exacerbating early-career researchers’ greatest concerns	Blog	UK	Reporter
33	17/09/2020	COVID-19: less than 1 in 4 staff feel safe returning to campus	News	Global	Reporter
34	18/09/2020	UK academics ‘at breaking point’ over shift to online teaching	News	UK	Associate Professor
35	01/10/2020	Asking staff to teach both online and face to face will tear them apart	Opinion	UK	Lecurer
36	09/10/2020	The pandemic is not an excuse to kill off the arts and humanities	Blog	UK/USA/AUS	Former academic and artist
37	29/10/2020	Heroism should not be part of the academic job description	Opinion	Global	Rankings editor and international reporter
38	23/11/2020	Universities ‘failing to support’ mental health first aiders	News	Global	Lecturer
39	08/12/2020	UK universities axe thousands of jobs during pandemic	News	UK	Dean
40	10/12/2020	Will COVID-19 cure the two-body problem?	Features	UK	Professor
41	10/12/2020	Men ‘worse at coping with disruption to research’ during pandemic	News	Global	Data Editor
42	06/01/2021	Strathclyde gives staff Fridays off during latest lockdown	News	UK	Reporter
43	11/01/2021	Academics demand v-cs and ministers rethink ‘key worker’ status	News	UK	Reporter
44	15/01/2021	Lost your job? How to turn COVID pressure into an opportunity	career	Global	Reporter
45	22/01/2021	Positives and pitfalls: what is good leadership in a crisis?	Opinion	Global	Reporter
46	28/01/2021	Staff or students? PhD students face worst of both worlds in lockdown	Blog	UK	Reporter
47	29/01/2021	Female academics bounced back in publishing as lockdowns eased	News	Global	Data Editor
48	02/02/2021	Union condemns Liverpool’s ‘rank and yank’ science cuts plan	News	UK	Assistant professor
49	04/02/2021	Staff say online teaching means more work and worse mental health	News	Global	PhD student
50	04/02/2021	Times Higher Education’s Digital Teaching Survey results	Features	Global	Data Editor
51	04/02/2021	From hurried to heroic	Opinion	Global	Reporter

The rest of this article presents and discusses the core themes identified through analysis. We use extracts from THE articles to evidence and illustrate the claims made. Each extract is followed by the article number to allow the reader to identify the exact source from Table 2.

## Findings

The analysis of Times Higher Education (THE) articles revealed a variety of issues that the COVID-19 pandemic has accelerated in academic work and higher education practices more broadly. The key examples relate to academic workload and wellbeing, casualization, intensified managerialism and gender and ethnic inequalities (see Table 1), and these form the core focus of the discussion that follows. However, it is important to note that a small number of articles (*n* = 9) highlighted the opportunistic side of the COVID-19 pandemic which we want to acknowledge before moving on to the main discussion. Such articles tended to frame the COVID-19 crisis as an opportunity to reform the UK higher education sector which would ultimately address the structural issues highlighted in the articles. Statements such as ‘The pandemic should offer us an opportunity for reflection and a moment to introduce radical change as our lives necessarily change’ (Article 17) and ‘The unthinkable has already become reality. The only question that remains is how our collective response will evolve to confront it’ (Article 22) illustrate such approach. Interestingly, these positive views of the crisis were promoted by senior university management. At an academic level, however, there was an acknowledgment of increased collegiality for developing student-centred practices, reflecting what Leask (2020: 1388) phrases as ‘embracing the possibilities of disruption’.

There is a shared feeling that we are all in this together and our colleagues have shown how supportive and adaptable they can be at such short notice. We have been trying to help each other out with best practice and lessons learnt during this time of unprecedented change (Article 3)

These positive reflections of the COVID-19 crisis, however, received little attention in the articles analysed. Our particular focus will now turn to the main challenges highlighted by the pandemic to offer what Marginson (2020: 1395) has vividly described as ‘a mirror of sorts, a means of common reflexivity’. As the main readership of THE magazine is academics, it is no surprise that the primary focus of the articles relates to academic workers and their experiences of the institutional life. We will start by outlining the most prevalent theme related to workload and wellbeing and conclude with the themes related to particular academic groups—women and ethnic minority staff—who are described as the most affected by the pandemic.

### Workload and Wellbeing

A large number of articles (*n* = 34; 67%) raised significant concerns for academic workload and wellbeing during the COVID-19 crisis. This is hugely important as emerging research and scholarship have primarily emphasized the mental health and wellbeing effects of the pandemic on students (*cf*. Aristovnik et al. 2020; Son et al. 2020). It was common for the articles to draw on recent research evidence to explain the severity of the issue. The surveys included the joint survey by the Student Mental Health Research Network and Vitae with 4800 doctoral and early career researchers (Vitae 2020) cited in the Article 10, THE Global Survey with 1195 academics and HE professionals worldwide (McKie and Basken 2020) cited in the Article 33, and THE Digital Teaching Survey with 520 academics from 46 countries (Prynne 2021) cited in the Articles 49 and 50. The survey evidence was used by THE reporters to comment on the effects of COVID-19 on academic workload and wellbeing, leading them to argue that the increased workload plays a major role in academics’ mental health and wellbeing.

In addition to an evidence-based approach taken by reporters whenever possible, the articles attempted to make academic voices heard, either by publishing academics’ opinion essays on workload and issues experienced, or including their commentary in various articles composed by THE editors/reporters:

University staff have said that while they feel conflicted about sending their children into schools given the strain on resources, the pressure to keep up with their workloads has led them to feel it is their only choice. (Article 43)

COVID resilience is being pursued at the expense of our resilience. We are being asked to teach double or triple what we were expecting, to cover unfamiliar material at short notice, using new methods, in collaboration with other people, all at personal risk to ourselves and our families. We are denied the choices and flexibility we are compelled to provide for our students, despite being more vulnerable to the virus. (Article 35)

The examples above highlight that it is the shift to online teaching and managing work and family life during the lockdown periods that have put particular pressure on academics’ workload. It is the intersection between the individual and social responsibility in responding to the pandemic (Marginson 2020) that has caused tensions in academic experience. The sense is that academics feel that they are being left to their own resources when dealing with increased work and family demands during the pandemic, having very little if any institutional understanding or support. This is also where the gender inequalities become pertinent as will be discussed later in this paper (see the section ‘Gender and ethnic inequalities’).

### Marketization of Higher Education and Intensified Managerialism

When discussing the workload issues and stress that academics were experiencing, the articles made a link with continuing effects of marketization and managerialism in British higher education. This indicates that the COVID-19 pandemic is not the root cause for the workload and wellbeing issues experienced, but such problematic experiences have been shaped by the continuing effects of marketization in UK higher education that have been widely covered by existing research (*cf*. Collini 2012; McCaig 2018; Moss 2012). In fact, the analysed articles demonstrate that the COVID-19 pandemic accelerated the market forces in the sector, and such intensified managerialism focused on the market survival of universities has resulted in the lack of positive institutional responses to the workload challenges academics were experiencing. It once again places responsibility onto the individual in coping with the crisis (Marginson 2020). THE outlet promoted the bleak future for UK universities. By citing the analysis by the Institute for Fiscal Studies (*cf*. Drayton and Waltmann 2020), THE editor emphasized the financial risks to UK universities: ‘About a dozen UK universities face going bust in the long run as a result of the COVID-19 crisis if they do not get a government bailout or help with their debts, new research has suggested’ (Article 23).

When addressing the financial outlook of the UK higher education sector, the articles introduced and critiqued various approaches taken by universities when mediating the effects of COVID-19 on their market position. The examples below relate to the University of Roehampton and the University of Cambridge responses, respectively:

The measures include: acceleration of work to generate new sources of income, such as new academic programmes; staff recruitment freeze; suspension of the senior and professorial pay review; voluntary severance and a voluntary flexible employment scheme; immediate salary reduction for the vice-chancellor and most senior staff. (Article 8)

A university spokesman said: ‘The University of Cambridge is not currently contemplating redundancies. We are doing everything in our power to support our staff and students during this public health crisis. The vice-chancellor shared with staff and students a range of scenarios in which the very worst-case would see a long-lasting global economic and health crisis that might severely affect the operation of the university and necessitate a range of unpalatable choices. These are planning scenarios, not predictions.’ (Article 11)

Such examples above outline the concerns for academic job security and staff redundancies which are portrayed as the likely scenario for many British universities. While articles produced by THE reporters/editors were primarily focused on future speculations, more critical voices on existing institutional practices were presented by an anonymous academic (Article 35) and a former academic (Article 36). It is likely that their less direct author positioning provided greater freedom for critique:

As a lecturer, I am ostensibly contracted to teach, research and administrate. COVID-19 has changed all that. For now, teaching takes precedence. Against the backdrop of a voluntary redundancy scheme, research has been explicitly relegated and all research budgets removed. This deficit will apparently be recovered in the future—although, for many temporary staff, that future does not exist, of course. (Article 35)

Some of the institutional responses seen in the UK, US and Australia bear the hallmarks of the Machiavellian manoeuvres that Littlefinger endorses, with the swingeing cuts at some universities going far beyond what is necessary, even amid a global pandemic. (Article 36)

When tracing the effects of the COVID-19 crisis and who the market-driven institutional approaches are likely to affect, the importance of themes on casual academic staff and gender and ethnic inequalities emerged. The rest of this paper problematizes the unequal effects of the COVID-19 pandemic on these groups in particular.

### Workforce Casualization and Precarity

It is evident from the analysis that the articles placed a significant emphasis on the COVID-19 risks of casual academic staff. Such problematization is unsurprising as it is widely known that academic work worldwide, but particularly in a market-driven system in the UK, has become insecure where ‘precariousness rather than security’ defines academic life and career progression (Gill and Donaghue 2016: 92). The UK University and College Union (UCU) (2020b) indicates that there are nearly 70,000 university teaching staff (primarily early career staff) working based on ‘atypical’ or so-called casual arrangements that reflect in hourly paid work on the lowest contract levels with no job security, sickness cover or holiday pay. It is likely that most undergraduate teaching is conducted by staff with short-term or bank contracts (UCU 2016). Gill and Donaghue (2016) even argue that higher education is one of the most casualized sectors of employment in the UK, second only to the hospitality industry.

Unlike with other themes identified through thematic analysis, the emphasis on casual academic staff became clearly evident from the article titles (see Table 2). The keywords in titles included a variety of related terminology, *e.g.* ‘fixed-term staff’, ‘casual contracts’, ‘higher education employment practices’ and ‘job losses’. When engaging with the issues, the reporters cited research and statistical evidence, ranging from recent surveys to the reports produced by the UCU. Interestingly, the survey evidence tended to come from the same sources as cited in many other THE articles (see the section ‘Workload and wellbeing’), demonstrating how THE outlet grounds itself in particular evidence and discourse communities, often produced by their own franchise as with THE Global Survey and THE Digital Teaching Survey. The examples of such interdiscursivity (Fairclough 1993) are evident from the examples below that use the research and report evidence to emphasize job security and continuing exploitation of casual academic staff:

Casual staff felt particularly abandoned. One objects that they were ‘not paid to undertake training in various new digital tools, whereas permanent staff attended training in their normal working hours’ [citing THE Digital Teaching Survey]. (Article 50)

Just one in 10 UK early career researchers whose contract is ending this year reports having received extra funding given the COVID-19 crisis, according to new survey results [citing the Student Mental Health Research Network and Vitae survey]. (Article 10)

If these cuts materialise, and were replicated across the UK sector, the number of fixed-term contracts not being renewed could run into the tens of thousands—potentially up to 30,000—the UCU claimed. (Article 26)

As British universities primarily rely on tuition fees for income, the sector has witnessed severe massification where the student numbers in the UK have reached over 2.5 million students in 2019/20 (HESA 2021). To improve the staff-student ratio that is essential for coping with a massified higher education sector, universities employ large numbers of casual teaching staff. If there are any risks to student numbers, as many of the analysed articles indicated, the casual staff will be among the first groups to lose their jobs.

### Gender and Ethnic Inequalities

As regards further inequalities that COVID-19 has accelerated, the articles (*n* = 12; 24%) mentioned the effects of the pandemic on female and ethnic minority academics. The phrases such as ‘Now, with almost all of us working from home and schools closed around the world, the burden of these responsibilities—particularly childcare—falls heavily on women’ (Article 9), and ‘Many of the female academics Times Higher Education spoke to said they felt “in limbo” (Article 19) were common to highlight how the COVID-19 pandemic has placed female academics in a disadvantaged position. While the articles emphasized the workload issues and balancing home/work responsibilities (as discussed in the section ‘Workload and wellbeing’), there was a further focus on COVID-19 effects on research work in particular. The following quotes draw on THE initiated study compiled by Digital Science, a London-based data analysis company which uses the Dimensions publication database to analyse over 60,000 journals across disciplines for a variety of publishing patterns during COVID-19, including gender representation (*cf.* Hook and Porter 2020):

The analysis shows that the proportion of accepted papers with a female first author dipped below the historical trend for submissions made in March, April and May. (Article 18)

Women’s research output appears to have bounced back in the latter part of 2020 as the lockdowns that closed schools and nurseries in many parts of the world were eased, new data suggest. (Article 47)

A further example draws on the project led by Harvard University and Northwestern University that surveyed over 45,000 staff on the effects of the COVID-19 pandemic on academic research in the US and Europe (*cf*. Myers et al. 2020):

Scientists with at least one child aged 5 or younger have seen their research time fall by 17 per cent during the coronavirus pandemic, with women more heavily affected. (Article 25)

As with other themes and the common discursive style of THE outputs, the coverage of gender and ethnic inequalities were dominated by research-informed articles, written by THE reporters with some occasional commentary from academics. However, a more radical account of gender inequalities is presented by a female academic in an opinion piece and using a metaphor of ‘baby penalty’:

Even in pre-coronavirus times, the “baby penalty” was a harsh reality of academic life. In the “new normal”, it seems, the situation is even worse. You can have either a job or a child. If you want both, be ready to sacrifice your physical and mental health. (Article 28)

Such a metaphoric account captures how childcare responsibilities, often accompanied by homeschooling during national lock downs, place female academics in a further disadvantaged position.

Ethnic inequalities were covered less frequently (n:4) compared to gender related issues (n:8); however, these are mentioned in relation to risks to casualized contracts (see the section ‘Workforce casualization and precarity’). The examples below illustrate the emphasis on ethnic minority staff and their job security. While the first example draws on the marking boycott organized by casualized staff at Goldsmiths, University of London (see more from Goldsmith’s Students’ Union 2020), the second is built around the UCU commentary that provided an authoritative voice to the issue at focus.

According to campaigners, this will result in the university losing a large proportion of its BAME teaching staff. ‘Figures we have collected suggest around 75 per cent of those being laid off are from a BAME background’. (Article 16)

Cuts to fixed-term roles have triggered concerns for racial equality, since 42 per cent of black and ethnic minority academics in the UK are on a fixed-term contract, compared with 31 per cent of their white colleagues. (Article 26)

These examples above and the evidence-informed approach taken by THE outlet demonstrate how the COVID-19 pandemic is likely to have a disproportionate effect on already marginalized groups in academia. While the issues of workload and intensified managerialist approaches will influence all staff working in British universities, the effects on female, ethnic minority, and early career academics cannot be underestimated.

## Discussion

The accounts presented from THE offer little respite from what has become a consensual view of the deleterious effects of UK universities’ response to the pandemic on the professional and personal lives of academics. They complement research which has revealed that academics are experiencing severe work intensification as they move entirely to online provision and an escalated pastoral role in response to the academic and social and emotional demands and mental health needs of students (Aristovnik et al. 2020); cognate disruption to work–life balance (Watermeyer et al. 2021), and struggles related to balancing the home-schooling needs of children (and home-caring needs of other dependents) and the pastoral care of students (Tarman 2020).

Endemic to these representations of *pandemia* is the refusal or be that inability of university leadership to adequately acknowledge and compensate for the harm visited upon their academic flock as a consequence of an unfaltering commitment to neoliberal reform. Compassionate and/or empathetic leadership is conspicuous for its absence, as is any evidence that academics’ struggles under lockdown could or would have been pre-empted, isolated, or in any better way, mitigated or resolved. Instead, we find a dominant representation of university leadership, in crisis containment mode, focused almost exclusively on matters of economic concern—and more tacitly, commercial exploitation—and a persistence with ‘business as usual’ despite the manifest proliferation of attendant social inequities (see for instance the section ‘Marketization of higher education and intensified managerialism’). This is an orientation we would argue that has been embedded with disruption in UK universities prior to the pandemic caused by lengthy industrial action (*cf.* UCU 2021).

Yet while these representations are far from flattering, we would suggest that their cumulative contribution is more than just the censure of compassionless leaders. Instead, we propose that *pandemia* elucidates the scale of universities’ market collusion and analogously the extent to which university leaders have evolved into dispassionate corporate functionaries, whose primary role is as guarantor for the market competitiveness and economic viability of the institutions they steward. Perhaps, therefore, in the milieu of higher education’s neoliberalization and related swing to performance management, audit culture, and the privileging of private gains over public goods, humanistic expectations of university leadership are unrealistic and misplaced. The contribution of COVID-19 in our estimation has been to further galvanize and normalize this shift.

A distinction, however, is necessary. COVID-19 is not the originator but the *accelerator* of higher education’s corporate migration and academics-related occupational distress. The moral impunity of university leadership, in the current context, is not so much, therefore, a revelation—as some of these media pieces intimate—but a new chapter within a recent history of responsibilization (Dougherty and Natow 2019) in universities and what Calhoun (2006) has called ‘the privatization of risk’, stimulated by neoliberal reform and what Brown (2015) calls ‘neoliberalism’s stealth revolution’. It is UK higher education’s transformation into a (quasi)market system that has caused academics to become responsible for both their productive capacity and professional resilience; the latter never more so necessary in an occupational field dominated or be that contaminated by hyper-competitiveness and the prevalence of individualistic and careerist behaviours, that are enacted interchangeably as self-preservation and self-promotion. Academics in the UK, as mostly everywhere, are accordingly recognizable as an occupational precariat, largely unshielded from unfavourable changes to work practices and conditions other than by the critical (and sometimes ‘soft’) solidarity of trade union membership. Their exposure to work intensification and exploitation (Angervall and Beach 2018; Sukarieh and Tannock 2019) and an unequal work-life balance; workplace harassment (Henning et al. 2017); bullying (Zabrodska and Kveton 2013), and microaggressions (Sue 2010); work inequalities (Niedzwiedz et al. 2020; Minello et al. 2021; Yildirim and Elsen-Ziya 2020); and the threats of redundancy have become constant features of their precarization, which in the COVID-19 context has become all the more apparent and debilitating (Kınıkoğlu and Can 2021), if normalized.

In having prioritized the financial efficacy of universities through the pandemic, academics are seen to have become an increasingly vulnerable and non-agentic constituency, in the latter case especially where a rationalization of leadership as crisis-management presents opportunities for non-democratic and non-consultative forms of institutional governance. As one THE opinion piece commented, “COVID resilience is being pursued at the expense of our resilience.” (Article 35) Such then is the extent of academics’ current vulnerability, particularly as it relates to deteriorations in their physical and mental health linked to a transition to remote working, work intensification and of course, redundancy (Bodin 2020), which makes claims to self-responsibilization and managerial disinterest in matters of health and wellbeing increasingly difficult to advocate or tolerate. Unpalatable as well are the pretensions of managerial interest and investment in staff health and wellbeing, reported in THE as the masquerade of rest and recuperation days offered without any corresponding workload reduction and amidst significant work intensification.

The ubiquity of work-based concerns represented in THE’s reporting of the impact of the pandemic on UK higher education suggests, therefore, a necessary and long overdue step-change in the frail relationship between university leadership and an academic rank-and-file, and a permanent detour from performance to person-centred management. However, realizing the potential of benevolent—and *socially* less *competitively* accountable—university leadership that might address and correct many of the social wrongs of neoliberal reform accelerated and accentuated by the pandemic may be less than straightforward. This may be especially so where a transition from campus-based to remote working, has at least in the present term, amplified a sense of ‘us and them’, of institutional disconnect and distrust. The physical decoupling of academics from university campuses has no doubt heightened feelings of alienation and disenfranchisement from institutional decision-making, further fractured a sense of professional belonging, yet concurrently empowered a managerial sleight of hand.

A transition to remote working has also brought about the increased (if unequally experienced) flexibilization of academic labour (Smyth et al. 2020). But so too has it augmented the privatization of risk and ‘entrepreneurial self-government’ (Dardot and Laval 2013) that reproduces and extends precarization; the unequal experience of which is no more so apparent than in the way remote working has disadvantaged the research productivity of female academics, and more broadly those with caring responsibilities (Myers et al. 2020), in addition to those whose terms of employment are weakest (Byrom 2020). The flexibility of remote working also comes at a cost to academics’ autonomy, freedom and longevity where technology is appropriated for purposes of surveillance (Bain et al. 2020), measurement (Williamson 2021) and even automation (Mirrlees and Alvi 2020). Any claims to freedom won by physical decoupling from campuses may be at best superficial and easily lost where digital resettlement unleashes the datafication of all aspects of academic labour that incentivizes new forms of digital performativity (Lupton et al. 2017) and engenders an oppressive form of ‘presenteeism’ and institutional shackling—an ‘electronic panopticon’ (Fernie and Metcalf 1998). The cost implications of universities going digital may similarly disproportionately impact certain groups, with human resource cost-savings hitting hardest those on insecure contracts; those whose teaching provision may be efficiently translated and updated online; and those employed in lower ranked institutions that may not weather the digital storm.

## Conclusion

The financial instability that characterized the UK’s higher education sector before the onset of the pandemic (McCaig 2018) has worsened as its disruptive effects have deepened. Much has already been documented in terms of the financial challenges presented by the pandemic to universities and income loss across universities’ catalogue of services (Blankenberger and Williams 2020; El Masri and Sabzalieva 2020) and more now is emerging in terms of the threat posed by private sector EdTech entities to universities’ market dominance in higher education provision (Mirrlees and Alvi 2020; Williamson and Hogan 2020). With prognoses of a potential permanent digital resettlement for higher education in certain country jurisdictions accompanied elsewhere, the UK particularly, with hesitancy concerning universities’ long-term organizational approach to taught delivery, commercial EdTech companies and transnational digital policy advocates have a lot to gain. The advance of higher education’s digitalization should also necessarily be understood in reference to global changes in the organization of work, and what is variously dubbed the fourth industrial revolution (Schwab 2017) and/or the era of the ‘digital economy’ (Bukht and Heets 2017). These are framings that both disrupt the preeminence of economic determinism in rationalizing the purpose and governance of universities and their contribution to labour market needs, and sow doubt of their continued relevance, where they are unable to keep pace with technological accelerations; accelerations further propelled by the pandemic. The potential for major reconfiguration of the higher education marketplace is consequently high, if it is not already under transition.

Such a perceived threat to universities’ monopolistic grip over higher education will no doubt have profound consequences for the lived experiences of academics and what we would suggest as the further intensification of universities’ neoliberal reform. Where digitalization causes the global higher education marketplace to become even more competitive, we would anticipate the further intensification of performance management and evaluation regimes within universities. The consequence of this happening will likely result in an even more inhospitable, flexibilized and analogously precarious work environment and further occupational jeopardy for academics (as ‘gig’ workers) as the transfer of weight of responsibilization to them augments and where university leadership is further blindsided by the positional hunt. There are of course other related concerns we have already highlighted related to pedagogical automation and the potential self-inflicted obsolescence of academics as digital journey(wo)men—links within a ‘chain or precarity’ (Montoya and Perez 2018)—and the dichotomy of their digital upskilling and pedagogical deskilling, and the ethical implications of ‘offshoring’ to online platforms.

Various grievances reported in the higher education trade press of the pandemic on the governance of universities and (re)organization and experience of academic life, would seem to be no passing trial. Instead with, in part,^2^ a grim recognition of the implausibility of a return to a pre-COVID status-quo, we surmise that the precarization of academic labour and cognate forms of occupational distress will distend, while managerialism will prosper and so too the fortification of the university as a neoliberal edifice. Collective and critical deliberations on the future of the university, led by academics as activists, are nevermore so urgent and necessary if this course is not to become a *fait accompli*. The university’s future cannot be left to its leadership if a purge of the precariat is to be avoided. The deprivatization of risk and collective responsibilization of academics as a *self-leading* herd must be grasped.

## Footnotes

1 For more information on THE World University Rankings, please visit: https://www.timeshighereducation.com/world-university-rankings/2021/world-ranking#!/page/0/length/25/sort_by/rank/sort_order/asc/cols/stats.

2 We *are* cognizant of the many affordances of digital pedagogies.

## References

[CR2] Agasisti T, Soncin M (2021). Higher education in troubled times: on the impact of COVID-19 in Italy. Studies in Higher Education.

[CR3] Angervall P, Beach D (2018). The Exploitation of Academic Work: Women in Teaching at Swedish Universities. High Educ Policy.

[CR4] Aristovnik A, Keržič D, Ravšelj D, Tomaževič N, Umek L (2020). Impacts of the COVID-19 pandemic on life of higher education students: A global perspective. Sustainability.

[CR5] Bain P, Taylor P, Mulvey G and Hyman J, (2020). Work Organization, Control and the Experience of Work in Call Centres. Work employment and Society, volume 16, Issue 1. 10.1177/09500170222119281

[CR6] Blackmore J (2020). The carelessness of entrepreneurial universities in a world risk society: a feminist reflection on the impact of COVID-19 in Australia. Higher Education Research & Development.

[CR7] Blankenberger B, Williams AM (2020). COVID and the impact on higher education: The essential role of integrity and accountability. Administrative Theory & Praxis.

[CR8] Bodin M (2020). University redundancies, furloughs and pay cuts might loom amid the pandemic, survey finds. Nature.

[CR9] Braun V, Clarke V (2006). Using Thematic Analysis in Psychology. Qualitative Research in Psychology.

[CR10] Braun V, Clarke V, Cooper H (2012). Thematic Analysis in *Handbook of Research Methods in Psychology*. Vol 2: Research Designs.

[CR11] Braun V, Clarke V (2013). Successful qualitative research: A practical guide for beginners.

[CR12] Brown W (2015). Undoing the demos: Neoliberalism’s stealth revolution.

[CR13] Bukht R, Heeks R (2017). Defining, conceptualising and measuring the digital economy. International Organisations Research Journal..

[CR14] Byrom. N. (2020). *COVID-19 and the Research Community: The challenges of lockdown for early-career researchers*. Available from: https://elifesciences.org/articles/59634 10.7554/eLife.59634 PMC7292644 32530421

[CR15] Calhoun C (2006). The privatization of risk. Public Culture.

[CR16] Collini S (2012). What are universities for?.

[CR17] Collins V, Rothe DL (2019). The violence of neoliberalism: Crime, harm and inequality.

[CR18] Dardot P, Laval C (2013). After Althusser: The Question of Marx’s Contemporaneity. Cités.

[CR19] de Boer H (2021). COVID-19 in Dutch higher education. Studies in Higher Education.

[CR20] Dougherty KJ, Natow RS (2019). Performance-based funding for higher education: how well does neoliberal theory capture neoliberal practice?. Higher Education.

[CR21] Drayton, E., and Waltmann, B. (2020). *Will universities need a bailout to survive the COVID-19 crisis? Briefing Note*. Available from: https://www.ifs.org.uk/publications/14919

[CR22] El Masri A, Sabzalieva E (2020). Dealing with disruption, rethinking recovery: Policy responses to the COVID-19 pandemic in higher education. Policy Design and Practice.

[CR23] Erickson M, Hanna P, Walker C (2020). The UK higher education senior management survey: a statactivist response to managerialist governance. Studies in Higher Education.

[CR24] Fairclough N (1993). Critical Discourse Analysis and the Marketization of Public Discourse: The Universities. Discourse and Society.

[CR25] Fernie, S. and Metcalf, D. (1998). *(Not)Hanging on the telephone: Payments systems in the new sweatshops*. Centre for Economic Performance: London School of Economics.

[CR26] Gewirtz S, Cribb A (2013). Representing 30 years of higher education change: UK universities and the Times Higher. Journal of Educational Administration and History.

[CR27] Gill R, Donaghue N (2016). Resilience, apps and reluctant individualism: Technologies of self in the neoliberal academy. Women's Studies International Forum.

[CR28] Goldsmiths Students’ Union (2020). *Marking boycott statement*. Available from: https://www.goldsmithssu.org/news/article/6013/Marking-boycott-statement/

[CR29] Harvey D (2005). A Brief History of Neoliberalism.

[CR30] Henning MA, Zhou C, Adams P (2017). Workplace harassment among staff in higher education: a systematic review. Asia Pacific Educ. Rev..

[CR31] HESA (2021) *Higher Education Student Statistics: UK, 2019/20 - Student numbers and characteristics*. Available from: https://www.hesa.ac.uk/news/27-01-2021/sb258-higher-education-student-statistics/numbers

[CR1] Hook, D., and Porter, S. (2020). *How COVID-19 is changing research culture*. Available from: https://digitalscience.figshare.com/articles/report/How_COVID-19_is_Changing_Research_Culture/12383267

[CR32] Kınıkoğlu CN, Can A (2021). Negotiating the different degrees of precarity in the UK academia during the COVID-19 pandemic. European Societies.

[CR33] Leask B (2020). Embracing the possibilities of disruption. Higher Education Research & Development.

[CR34] Lupton D, Mewburn I, Thomson P (2017). The digital academic: Critical perspectives on digital technologies in Higher Education.

[CR35] Marginson S (2020). The relentless price of high individualism in the pandemic. Higher Education Research & Development.

[CR36] McCaig C (2018). The Marketisation of English Higher Education: A Policy Analysis of a Risk.

[CR37] McKie, A., and Basken, P. (2020). COVID-19: less than 1 in 4 staff feel safe returning to campus*. Times Higher Education*. Available from: https://www.timeshighereducation.com/news/COVID-19-less-1-4-staff-feel-safe-returning-campus

[CR38] Metcalfe AS (2021). Visualizing the COVID-19 pandemic response in Canadian higher education: an extended photo essay. Studies in Higher Education.

[CR39] Minello A, Martucci S, Manzo LKC (2021). The pandemic and the academic mothers: present hardships and future perspectives. European Societies.

[CR40] Mirlees T, Alvi S (2020). EDTECH INC: Selling, automating and globalizing higher education in the digital.

[CR41] Moss, M. (2012). *Education and its Discontents. Teaching, the humanities, and the importance of a liberal education in the age of mass information*. Plymouth: Lexington Books.

[CR42] Myers KR, Tham WY, Yin Y (2020). Unequal effects of the COVID-19 pandemic on scientists. Nat Hum Behav.

[CR43] Niedzwiedz CL, Green MJ, Benzeval M (2020). Mental health and health behaviours before and during the initial phase of the COVID-19 lockdown: longitudinal analyses of the UK Household Longitudinal Study. J Epidemiol Community Health.

[CR44] Olssen M, Peters MA (2005). Neoliberalism, higher education and the knowledge economy: from the free market to knowledge capitalism. Journal of Education Policy.

[CR45] Parr. C. (2014). Imperial College professor Stefan Grimm ‘was given grant income target’. *Times Higher Education*. Available from: https://www.timeshighereducation.com/news/imperial-college-professor-stefan-grimm-was-given-grant-income-target/2017369.article

[CR46] Pells, R. (2018). Lecturer’s suicide a ‘wake-up call’ on overworking in academia. *Times Higher Education*. Available from: https://www.timeshighereducation.com/news/lecturers-suicide-wake-call-overworking-academia

[CR47] Perez M and Montoya A , (2018). The Unsustainability of the Neoliberal Public University: Towards an Ethnography of Precarity in Academia. Revista de Dialectología y Tradiciones Populares, vol. LXXIII, n.o 1, pp. A1-A16, enero-junio 2018, ISSN: 0034–7981, eISSN: 1988–8457.

[CR48] Prynne M. (2021). Digital Teaching Survey special report. *Times Higher Education*. Available from: https://www.timeshighereducation.com/campus/download-digital-teaching-survey-special-report

[CR49] Raaper, R. & Brown, C. (2020). The COVID-19 pandemic and the dissolution of the university campus: Implications for student support practice. *Journal of Professional Capital and Community*. Online First. 10.1108/JPCC-06-2020-0032

[CR50] Raaper R, Olssen M (2016). Mark Olssen on neoliberalisation of higher education and academic lives: An interview. Policy Futures in Education.

[CR51] Schwab K (2017). The fourth industrial revolution.

[CR52] Smyth J (2017). The toxic university: Zombie leadership, academic rock stars and neoliberal ideology.

[CR53] Smyth C, Cortis N, Powell A (2020). University staff and flexible work: inequalities, tensions and challenges. Journal of Higher Education Policy and Management.

[CR54] Son, C., Hegde, S., Smith, A., Wang, X., & Sasangohar, F. (2020). Effects of COVID-19 on college students’ mental health in the United States: Interview survey study*. Journal of Medical Internet Research*, 22(9). 10.2196/21279 PMC7473764 32805704

[CR55] Stack M (2020). Academic stars and university rankings in higher education: impacts on policy and practice. Policy Reviews in Higher Education.

[CR56] Sue DW (2010). Microaggressions in everyday life: Race, gender, and sexual orientation.

[CR57] Sukarieh M, Tannock S (2019). Subcontracting Academia: Alienation, Exploitation and Disillusionment in the UK Overseas Syrian Refugee Research Industry. Antipode.

[CR58] Tamboukou M (2012). Truth telling in Foucault and Arendt: parrhesia, the pariah and academics in dark times. Journal of Education Policy.

[CR59] Tarman B (2020). Editorial: Reflecting in the shade of pandemic. Research in Social Sciences and Technology.

[CR60] THE (n.d.). *Times Higher Education. Helping the world’s universities to achieve excellence*. Available from: https://www.timeshighereducation.com/about-us#:~:text=Whether%20you%20are%20looking%20to,%2C%20rankings%2C%20jobs%20and%20data

[CR61] Thornton M (2013). The mirage of merit. Australian Feminist Studies.

[CR62] UCU (2016). *Precarious work in higher education: A Snapshot of Insecure Contracts and Institutional Attitudes*. https://www.ucu.org.uk/media/7995/Precarious-work-in-higher-education-a-snapshot-of-insecure-contracts-and-institutional-attitudes-Apr-16/pdf/ucu_precariouscontract_hereport_apr16.pdf

[CR63] UCU (2020a). UCU announces 14 strike days at 74 UK universities in February and MarchAvailable from: https://www.ucu.org.uk/article/10621/UCU-announces-14-strike-days-at-74-UK-universities-in-February-and-March

[CR64] UCU (2020b). *Survival guide for hourly-paid staff*. Available from: https://www.ucu.org.uk/media/4647/UCU-hourly-paid-survival-guide/pdf/ucu_hourlypaidsurvivalguide_jan20.pdf

[CR65] UCU (2021). *Action for USS*. Available from: https://www.ucu.org.uk/strikeforuss

[CR66] van Schalkwyk F (2021). Reflections on the public university sector and the COVID-19 pandemic in South Africa. Studies in Higher Education.

[CR67] Vitae (2020). Release of initial findings to sector following response to COVID-19 survey. Available from: https://www.vitae.ac.uk/news/vitae-news-2020/release-of-initial-findings-to-sector-following-response-to-covid-19-survey

[CR68] Wacquant L (2010). Crafting the neoliberal state: Workfare, prisonfare, and social insecurity. Sociological Forum.

[CR69] Watermeyer R (2019). Competitive accountability in academic life: The struggle for social impact and public legitimacy.

[CR70] Watermeyer R, Olssen M (2016). ‘Excellence’ and exclusion: The individual costs of institutional competitiveness. Minerva.

[CR71] Watermeyer R, Tomlinson M (2021). Competitive accountability and the dispossession of academic identity: Haunted by an impact phantom. Educational Philosophy and Theory.

[CR72] Watermeyer R, Crick T, Knight C, Goodall J (2021). COVID-19 and digital disruption in UK universities: afflictions and affordances of emergency online migration. Higher Education.

[CR73] Watermeyer, R., Chen, Z., and Ang, B. ‘Education without limits’: The digital resettlement of post-secondary education and training in Singapore in the COVID-19 era. Journal of Education Policy. (forthcoming)

[CR74] Watermeyer, R., Shankar, K., Crick, T., Knight, C., McGaughey, F., Hardman, J., Suri, V.R., Chung, R. and Phelan, D. ‘Pandemia’: A reckoning of UK universities’ corporate response to COVID-19 and its academic fallout. British Journal of Sociology of Education. (forthcoming)

[CR75] Williamson B (2021). Making markets through digital platforms: Pearson, edu-business, and the (e)valuation of higher education. Critical Studies in Education.

[CR76] Willamson, B. and Hogan, A. (2020). The edtech pandemic shock. Worlds of Education. Available from: https://www.worldsofeducation.org/en/woe_homepage/woe_detail/16856/the-edtech-pandemic-shock-by-ben-williamson-anna-hogan

[CR77] Yildirim MT, Elsen-Ziya H (2020). The differential impact of COVID-19 on the work conditions of women and men academics during the lockdown. Gender, Work and Organization..

[CR78] Zabrodska K, Kveton P (2013). Prevalence and Forms of Workplace Bullying Among University Employees. Employ Response Rights.

